# An alien parasite in a changing world – *Ashworthius sidemi* has lost its traditional seasonal dynamics

**DOI:** 10.3389/fvets.2023.1279073

**Published:** 2023-11-01

**Authors:** Jan Magdálek, Lucie Škorpíková, Christopher McFarland, Jaroslav Vadlejch

**Affiliations:** ^1^Department of Zoology and Fisheries, Faculty of Agrobiology, Food and Natural Resources, Czech University of Life Sciences Prague, Prague, Czechia; ^2^Department of Botany and Zoology, Faculty of Science, Masaryk University, Brno, Czechia; ^3^School of Biological Sciences, Institute for Global Food Security, Queen’s University Belfast, Belfast, United Kingdom

**Keywords:** invasive nematode, fallow deer, epidemiology, anthelmintic drug, temperature

## Abstract

A non-native nematode *Ashworthius sidemi* has emerged in captive fallow deer in Central and Eastern Europe over the last decade. Although this parasite has been spreading in the wild outside it’s native distributional range and colonising local European host species since the middle of the last century, limited information has been published on the seasonality of *A. sidemi* and its susceptibility to anthelmintics. To address this knowledge gap, we conducted a study to investigate seasonal dynamics of the non-native parasite in the current Central European climate conditions. We collected freshly voided faecal pellets at four-week intervals from February 2018 to February 2020 at a fallow deer reserve with a known history of *A. sidemi* presence. The faecal pellets obtained were pooled after each site visit (*n* = 25) and coprocultured to obtain the third stage larvae of trichostrongylid nematodes at monthly intervals. Total genomic DNA was extracted from the recovered larvae. Using real-time multiplex PCR, *A. sidemi* DNA was detected in 17 out of 25 larval samples (68% prevalence). During the monitoring period, the annual administration of ivermectin based premix (Cermix) took place in January 2018, 2019, and 2020, and additionally a mixture of rafoxanide and mebendazole (Rafendazol) was administered once in spring 2019. The probability of parasite presence was significantly influenced by the time since the drug administration (*p* = 0.048) and the mean temperature at the location (*p* = 0.013). Larval samples negative for *A. sidemi* were always identified shortly after the drug administration. However, rapid pasture contamination by the parasite eggs from two to three months after Cermix administration and within one month after Rafendazol administration suggest only a short-lived efficacy of both administered drugs. The abundance of *A. sidemi* DNA was positively affected by mean temperature (*p* = 0.044) and remained relatively stable throughout the monitoring period, with the highest peak in August 2018 and 2019. Pasture contamination with *A. sidemi* eggs occurred almost all year round, with the exception of the beginning of 2018, 2019, and 2020. These findings indicate adaptation of a non-native parasite to the current climatic conditions of the Czech Republic resulted in negligible seasonal patterns of parasite egg shedding.

## 1. Introduction

The fallow deer (*Dama dama*) is currently one of the most widespread cervids in the world, and despite being considered an allochtonous species in many ecosystems (1), its importance in agriculture is growing with the increasing interest in alternative sources of meat for human consumption. In Europe, the fallow deer is the second most farmed cervid after the red deer (2), and is now widely distributed both in hunting grounds and game reserves (3).

Like other ruminants, fallow deer is susceptible to infection with gastrointestinal (GI) nematodes. Although such infections are predominantly subclinical, they may have a significant impact on both the population dynamics of cervids and their individual fitness (4). The abomasal nematodes of the superfamily Trichostrongyloidea (trichostrongylids), especially *Ostertagia* spp. and *Spiculopteragia* spp. were traditionally considered to be the most important parasites in fallow deer (5–7); however, more recently there have been reports on emergence of an invasive nematode, *Ashworthius sidemi*, in captive fallow deer (8, 9). The alien parasite was introduced to Europe via sika deer in the late 19th century and subsequently disseminated among local cervid hosts, especially in Central and Eastern Europe (10–13). Currently, *A. sidemi* may be spread by migrating cervids and through further unintended human-mediated introductions (14, 15).

Transmission of *A. sidemi* occurs through accidental ingestion of infective larvae (L3) by a susceptible ruminant host when grazing. The endogenous development involves abomasal mucosa invasion and several larval molts to adult stages, which undergo sexual maturity and reproduction. Time elapsed between infection and shedding of nematode eggs may be delayed by arrested larval development (hypobiosis); the phenomenon occurs in response to unfavorable environmental and/or host conditions (16). Eggs are released with host faeces contaminating grazing pasture while their further fate, i.e., hatching, survival, development to the infective stage L3_,_ and their movement on a pasture, is strongly influenced by environmental factors, especially humidity, and temperature (17). Trichostrongylid infections thus show seasonal patterns, which are an outcome of environmental, host, and parasite factors that are variable within a year. A comprehensive understanding of how the nematode population fluctuates throughout the season is crucial for planning effective and sustainable parasite control. While the seasonal dynamics of trichostrongylid infections in ruminant livestock have been thoroughly studied for many decades (18, 19), only limited information are available for cervids.

Certain aspects of *A. sidemi* seasonal dynamics were outlined based on necropsies of several wild ruminant species during a hunting season in Poland (11). The authors concluded that transmission of *A. sidemi* occurs from June to September, while during the winter and following spring, the non-native nematode survives only as L4 larvae and sexually immature individuals. Similar results have been previously published (20); only larvae and juveniles of *A. sidemi* were recovered from the abomasa of sika deer and Maral deer during autumn and winter in the Russian Far East. In contrast to the above mentioned studies, only negligible importance of hypobiosis has been detected in the Czech Republic (CR), indicating a possible modification of seasonal patterns in *A. sidemi* (15, 21). Seasonal dynamics of trichostrongylids can differ based on the climate conditions in different regions and may also shift over time due to global climate changes (22, 23). Seasonal patterns of *A. sidemi* egg laying remain unclear, as a longitudinal study has yet to be conducted.

Management practices, such as anthelmintic treatment, can disrupt the seasonal patterns of GI nematodes by suppressing egg output (24, 25). While the use of anthelmintic drugs in wild ruminants is somewhat controversial (26), cloven-hoofed animals at game reserves and especially at farms are often kept in overcrowded conditions and may suffer from parasitic infections to a similar extent as ruminant livestock. Application of anthelmintic treatments to such animals is therefore justified (27). Hunting legislation in the CR allows the administration of anthelmintics in the controlled conditions of game reserves and farms throughout the year. However, the common practice is to apply anthelmintic treatments to the animals during the winter season when natural food sources are limited, and drug efficacy is at its peak. Two anthelmintic drugs in the form of medicated feed are certified for this purpose in the CR – Rafendazol, a mixture of rafoxanide and mebendazole, and ivermectin-based premix Cermix. The high efficacy of both these drugs against GI nematodes has been previously demonstrated in various species of cloven-hoofed animals (28, 29). Long-term annual administration of Cermix to fallow deer resulted in a gradual suppression of both the prevalence and the faecal egg counts (FECs) of strongylid nematodes (27). However, the efficacy of any anthelmintics against *A. sidemi* infection has yet to be published.

Monitoring the fluctuation of GI nematode populations is traditionally based on FECs; however, the vast majority of trichostrongylids (including *A. sidemi*) cannot be reliably distinguished based just on morphological identification of nematode eggs (30). A molecular method was proposed earlier that enables reliable *A. sidemi* identification based on larval DNA isolates (31). This is achieved by amplifying specific sequences found in the ITS1 and ITS2 regions of ribosomal DNA using a simple PCR technique. Later, a real-time multiplex PCR method was optimized, allowing the detection of the parasite in co-infection with multiple nematode species (including *A. sidemi*) and semi-quantitative estimation of parasite burden (32).

The current study aims to fill the knowledge gap concerning the seasonality of a non-native nematode *A. sidemi* in cloven-hoofed animals in the current climatic conditions of Central Europe. We conducted a two-year study in a fallow deer reserve under intensive breeding conditions and routine management practices including regular administration of anthelmintics. Molecular techniques were used to monitor *A. sidemi* seasonal dynamics in host fallow deer during the two-year period from February 2018 to February 2020.

## 2. Materials and methods

### 2.1. Study site and animals

**Figure 1:**
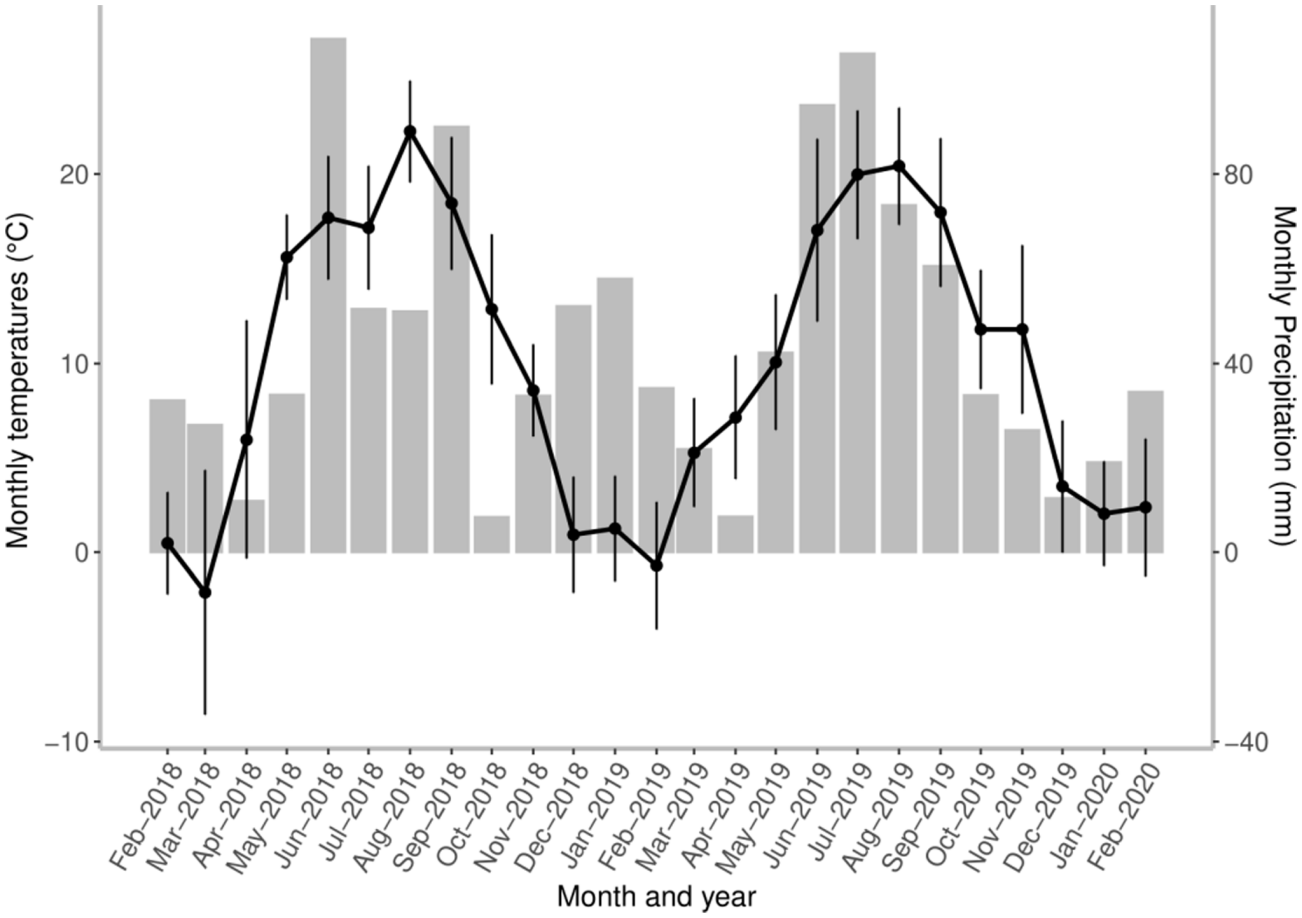
The course of environmental conditions shows local changes in mean monthly temperature (black points, standard deviation (SD) and total precipitation (grey bars) measured at the game reserve throughout the study period. All local weather information was obtained from the Czech Hydrometeorological Institute.

The seasonal dynamics of *A. sidemi* was monitored on a game reserve in the village of Budyně in the South Bohemian Region of the Czech Republic (49°8′ 48.39” N, 14°4′ 11.96″ E). According to the Köppen–Geiger climate classification (33) the study location is represented by a warm-summer humid continental climate found in much of Central Europe. This climate is characterized by four distinct seasons with seasonal temperature differences of mild to warm summers, long cold winters, and reduced precipitation (34). Meteorological data at the study location obtained from the Czech Hydrometeorological Institute are presented in Figure 1.

The game reserve occupies an area of approximately fifty hectares, mostly covered with perennial grassland and a narrow strip of sparse oak grove in the centre. A narrow stream runs through the reserve. A herd of approximately three hundred fallow deer in a balanced ratio of males and females were kept at the game reserve during the study period. Their diet consisted of year-round grazing, except during periods of continuous snow cover, supplemented by continuous feeding of concentrates. Additional feeding was carried out at a single common feeding site, where feed mixed with anthelmintics was also provided at timepoints throughout the grazing season.

### 2.2. Anthelmintic treatment

The anthelmintic drug Cermix (Biopharm – Research Institute of Biopharmacy and Veterinary Drugs, CR) containing ivermectin was administered annually in January 2018, 2019, and 2020 in the form of medicated feed. In addition, Rafendazol (Biopharm – Research Institute of Biopharmacy and Veterinary Drugs, CR), a mixture of rafoxanide and mebendazole, was applied once at the end of March 2019 by a game reserve owner. Dosage requirements for each anthelmintic were estimated according to the number of animals to reach the therapeutic dose recommended by the manufacturer, i.e., 0.2 mg of ivermectin and 10 mg of mebendazole per kilogram of live body weight. The application of anthelmintics was always preceded by a one-week adaptation to non-medicated concentrated feeds. Anthelmintics were administered on the feeding site in a mixture with concentrates at the prescribed ratio of one part drug to nine parts feed. The medicated mixture was administered according to the drug manufacturer’s instructions.

### 2.3. Sample collection

Freshly voided faecal pellets of fallow deer were collected throughout the game reserve at four-week intervals from 15th of February 2018 to 15th of February 2020 resulting in a total of 25 site visits. During each visit, faecal pellets from 20 animals were collected opportunistically in an attempt to cover the entire area of the reserve and to avoid repeated sampling of individuals. The herd was monitored through binoculars from a sufficient distance to avoid stressing the animals, while the collection of faeces was carried out immediately after their movement to another part of the game reserve. Logistical constraints meant that it was not possible to collect faeces from individual animals using rectal sampling. Furthermore, due to the size of the herd, it was challenging to identify individuals accurately, thus we could not provide information on the sex of the individuals relative to the faecal pellets collected. However, it was possible to differentiate the faecal samples from adult deer and young deer, based on the size of pellets. Faecal pellets were collected exclusively from adult deer as this age group within the herd was more likely to exhibit anthelmintic suppresion of *A. sidemi* egg output based on the anthelmintic administration method. Faeces were collected as soon as possible after defecation to avoid damage of the GI nematode eggs by freezing or desiccation. Faecal pellets collected from 20 individual animals at each site visit were pooled into a single 1 kg sample, stored in an open plastic box, and immediately transported to the laboratory for further processing. Clinical signs of disease were not observed in animals during the study period.

### 2.4. Parasitology

To examine the presence of *A. sidemi* in faecal samples, separate larval coprocultures were established from pooled faecal pellets collected at each sampling visit (*n* = 25) according to the methods of Hansen and Perry (35). Briefly, faecal material was homogenized, mixed with vermiculite and moistened, then placed in open microtene bags in an incubator for seven days at 27°C. During the incubation period, the coproculture was checked daily, homogenized by rubbing between the fingers to prevent mold formation, and moistened if necessary. At the end of incubation, the coproculture was transferred to the Baermann apparatus for the release of the L3s from the faecal material. After 24 h, the fluid containing the larvae was released into conical glass containers and decanted. The sediment formed by the larvae was subsequently purified by repeated Baermanization and stored at 4°C.

### 2.5. Molecular analysis

For each sample, total genomic DNA was extracted from L3s using the DNeasy Blood & Tissue commercial kit (QIAGEN, Hilden, Germany). Prior to DNA extraction, larvae were washed several times with sterile distilled water. The pooled L3s were then incubated at 56°C for 48 h in 180 μL ATL buffer and 20 μL proteinase K. Subsequent purification steps followed the manufacturer’s protocol. The DNA extracts were immediately stored at −20°C until further processing.

Molecular detection of *A. sidemi* DNA presence and relative quantity within L3 pools was based on triplex real-time PCR using TaqMan technology and consisted of detection systems for (i) *A. sidemi* (targeting ITS1 sequence), (ii) a calibration standard, and (iii) an internal amplification control (IAC), which were adopted from previous studies (32, 36).

The reaction mixture contained: 1× Luna Universal Probe qPCR Master Mix (New England Biolabs, Ipswich, MA, United States), 250 nM of each primer, 100 nM of FAM probe, 100 nM Cy5 probe, 200 nM of HEX probe, 0.4 U of Antarctic Thermolabile UDG (New England Biolabs, Ipswich, MA, United States), 1× 10^4^ copies of IAC plasmid, 5 μL of template DNA and nuclease-free water to complete 20 μL volume.

All samples were run in duplicate on a fluorometric thermal cycler CFX96 Real-Time PCR Detection System (Bio-Rad Laboratories, Hercules, CA, United States) with the following conditions: incubation step at 25°C for 10 min (carryover prevention), initial denaturation at 95°C for 2 min followed by 40 cycles of amplification with 95°C for 15 s for denaturation, and 57°C for 45 s for combined annealing and extension, with the collection of fluorescence signal at the end of each cycle. The data was evaluated using the software Bio-Rad CFX Manager 3.0 (Bio-Rad Laboratories, Hercules, CA, United States).

The calibration curve was constructed using serially diluted plasmid standard (5× 10^7^, 5× 10^6^, 5× 10^5^, 5× 10^4^, and 5× 10^3^ copies per μL) and was used to extrapolate the amount of target DNA of *A. sidemi* in each sample. Detailed information on the optimization and design of detection systems, primer and probe sequences, and quantitative data evaluation is provided in the publication (32).

### 2.6. Data analysis

We considered two dependent variables: (i) the presence or absence of *A. sidemi* DNA (coded as 1 or 0) and (ii) the relative quantity of *A. sidemi* DNA. We tested the effect of external conditions and anthelmintic treatment on each variable. Data analysis consisted of two steps. First, the effect of these factors on the presence of the target *A. sidemi* DNA was tested separately for each explanatory variable using simple logistic binomial regression. Then, the relative level of DNA identified in the positive samples was modeled using quasi-Poisson generalized linear regression.

The explanatory variables included the average daily temperatures (°C) and total rainfall (mm) measured four weeks prior to each sample collection and factor of treatment (weeks post administration) expressed as the number of weeks elapsed between the last application of the anthelmintic drug and the sampling date. *p* values ≤0.05 were considered significant. All statistical tests were performed in R 4.1.3 (37). Data are presented in graphs created using the ggplot2 R package (38).

## 3. Results

**Figure 2:**
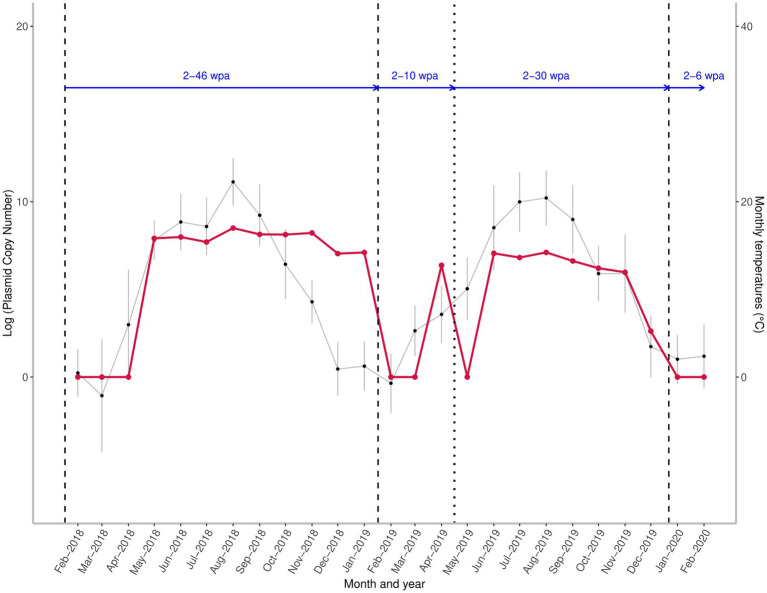
Seasonal variation in the quantity of the target A. sidemi DNA at a fallow deer game reserve between 2018 and 2020 relative to administration of anthelmintic and course of average monthly temperatures. Red line shows the fluctuation of parasite DNA levels expressed as the logarithm to base 10 (log) of the plasmid standard copy number. Vertical lines indicate the time of the Cermix (dashed) and Rafendazol (dotted) anthelmintic applications. Blue horizontal arrows represent the number of weeks elapsed since the last anthelmintic application (wpa – weeks post administration). Monthly average temperatures are denoted by black points.

The presence of *A. sidemi* DNA was molecularly confirmed in 17 out of 25 larval pooled samples (68%). The probability of target parasite DNA being detected in samples was positively (*p* = 0.048) affected by the time elapsed since anthelmintic administration (Table 1). The seasonal *A. sidemi* patterns detected within the study are presented in Figure 2. Samples collected during the first three visits, from mid-February to mid-April 2018 tested negative for *A. sidemi* DNA. The parasite was first detected fourteen weeks after the Cermix administration in May 2018. A similar pattern was observed in 2019; after the second application of Cermix in late January, the parasite was not observed for ten weeks, and the emergence of *A. sidemi* DNA was first detected in mid-April 2019, a month earlier than in the previous year. Furthermore, following the January application of Cermix in early 2020, the samples collected yielded negative results.

**Table 1:** Model estimates of logistic regression and quasi-poisson generalised linear regression (GLM) testing influence of temperature, rainfall and anthelmintic treatment on presence of *A. sidemi* DNA in all samples.

Factor	Presence/absence (logistic regression)		DNA level (GLM)	
	Coefficient ± SD	*p*-value	Coefficient ± SD	*p*-value
Mean temperature	0.280 ± 0.112	0.013	0.021 ± 0.010	0.044
Rainfall	0.044 ± 0.024	0.076	0.001 ± 0.002	0.606
Wpa	2.467 ± 1.250	0.048	0.037 ± 0.021	0.092

Analyses of relative quantity of *A. sidemi* DNA was performed on positive samples only. SD – Standard Deviation, Wpa – weeks post anthelmintic administration.

An interruption of the spring rise in target DNA level and a decrease to zero was observed in May 2019, two weeks after Rafendazol administration; however, over the next four weeks, there was a rapid recovery in measured DNA amount, and the amount of *A. sidemi* DNA detected in June 2019 exceeded the April 2019 values.

The probability of the presence of *A. sidemi* DNA was positively influenced by the mean temperature measured four weeks prior to sampling (Table 1). With the exception of April 2018 and May 2019, cooler temperatures below 5°C preceded the collection of all negative samples (Figure 2).

The abundance of target DNA detected in positive samples did not show high variation and was only slightly affected by the mean temperature prior to sampling (Table 1). Relative *A. sidemi* DNA quantities detected during the first season remained at a relatively consistent level from May to November 2018. The first noticeable decline in *A. sidemi* DNA relative quantity occurred between November and December 2018, but the parasite was still detected until mid-January 2019. Dynamics of *A. sidemi* DNA in larval samples, and thus pasture contamination throughout 2019, showed a similar pattern to the previous year; after a moderate decline in July 2019, a peak was recorded in August 2019, followed by a gradual decline during the autumn and winter.

No significant impact of precipitation on either the presence or the abundance of parasite DNA was observed in this study (Table 1). The median of *A. sidemi* DNA values detected in the 2019 samples was significantly lower compared to the previous year (*p* < 0.01). The maximum values detected in the summer 2019 were approximately equal to the minimum values of the positive samples in 2018.

## 4. Discussion

We determined the presence and relative quantity of *A. sidemi* DNA from L3s coprocultured from faecal samples collected from fallow deer in a game reserve over a two year study (2018–2020) in the CR. The amount of detected *A. sidemi* DNA varied between monthly sampling events, with multiple zero values observed during the winter and spring seasons. The absence of *A. sidemi* DNA from coprocultured L3 consistently appeared no later than two weeks after anthelmintic administration with Cermix or Rafendazol, suggesting a potential decrease in egg production or removal of adult worms in response to treatment. This finding was consistent with the reduction of strongylid FECs to zero values observed in mouflon from the second day after anthelmintic treatment with Cermix medicated mixture (29). Furthermore, a previous study investigating the seasonal dynamics of strongylids at a fallow deer farm in Poland, observed a temporary decrease in both prevalence and infection intensity after percutaneous administration of ivermectin; however, a resurgence of both characteristics occurred within the following two months (24). Similarly, the absence of the parasite during our survey persisted from one to three months following anthelmintic treatment.

The absence of *A. sidemi* DNA from coprocultured L3 during winter may reflect the natural seasonal patterns of the parasite. Previous post-mortem and coprological examinations of untreated cervids in various temperate regions have shown a lower prevalence and intensity of strongylid infection during the winter period (39, 40). Conversely, some studies have indicated higher prevalences and intensities of trichostrongylids in winter when supplementary feed is provided, suggesting the importance of winter feeding sites for transmission of parasites (41). Based on our results, we cannot definitively determine to what extent environmental conditions contributed to the observed winter reduction of *A. sidemi* DNA from coprocultured L3, as the probability of sample positivity may have been affected by both the time elapsed since anthelmintic treatment and the mean temperature. In the current study, we used published faecal culture protocols which were standardised at all sampling events (35). This technique is suitable for a wide range of GI nematode species; however, the survival rate and successful development of *A. sidemi* to the infective L3 larval stage in coproculture has not been examined. A molecular assay used in this study was previously found capable of identifying the parasite at input DNA levels as low as 5 pg (32). However, due to the assumed low infection intensity, we cannot exclude that the amount of DNA isolated from coprocultured L3 derived from faecal samples collected during the winter were not below the sensitivity threshold of the assay.

Differences in temperature at the study site may have caused variations in the emergence time of *A. sidemi* on pasture following anthelmintic treatment. In 2019, *A. sidemi* DNA was detected in mid-April (10 weeks post treatment application – wpa), whereas in 2018, it was not observed until May (14 wpa). This delay could be attributed to freezing temperatures persisting until March 2018. In contrast, the rapid reappearance of *A. sidemi* DNA four weeks after the administration of Rafendazol in May 2019 may have been the result of favorable conditions for larval development on pasture resulting in infection of grazing hosts, low efficacy of Rafendazol treatment or a combination of both. Potential sources of parasite infection in this herd may have been untreated individuals, especially juvenile hosts acting as a refugia for the parasite. Furthermore, the form of anthelmintic administration may lead to uneven dosage of drugs as the dominant individuals usually get first to the attractive medicated food, leaving weaker animals and especially the younger individuals, to consume remaining feed (42). According to Coop and Kyriazakis (43), the allocation of limited nutrient resources by the host prioritizes growth and reproduction over immunity to parasites. During pregnancy and lactation, both domestic and wild ruminants frequently exhibit periparturient rise (PPR), characterized by an increase of FECs (44, 45). Given that fawning on the monitored reserve occurred from May onwards, we cannot rule out reproducing females as a potentially significant source of *A. sidemi* during the spring and early summer. In August 2018 and, to a lesser extent, in 2019, we observed a slight peak in *A. sidemi* DNA levels, which approximately coincided with the weaning of early summer-born fawns. We did not collect data on juveniles in this study; however, this age category might also have an important role in pasture contamination later in the grazing season as they are exposed to high summer levels of infective larvae as naive hosts.

Contamination of the monitored pasture with *A. sidemi* is likely to have occurred rapidly rather than by a gradual process given the rise in *A. sidemi* DNA levels from coprocultured L3 from zero to near peak levels four weeks after anthelmintic application. This may have been influenced by high animal densities, which could facilitate the transmission of parasites (6). Similarly, rapid reinfection to levels equal to or higher than pre-treatment levels was observed in sheep within six weeks after drug administration (46).

Assessment of *A. sidemi* DNA levels from coprocultured L3 at multiple sampling events showed that the anthelmintic treatment had no significant impact on the long-term amount of DNA detected in the positive samples, suggesting a short-term effect of both anthelmintics used against the parasite. However, the levels of *A. sidemi* DNA detected in 2019 were significantly lower than those detected in 2018, despite environmental conditions being perceived as more favourable for larval development. Since there were no significant changes in the number of animals or herd structure (age or sex groups) between the monitored years, the repeated and frequent administration anthelmintics may have contributed to the decline of *A. sidemi* DNA detected over time. A previous study in a fallow deer reserve in the CR showed that repeated Cermix administration over seven years led to a gradual reduction in general strongylid infection intensity by 75% and complete elimination of *Haemonchus contortus* (27).

The findings from previous studies carried out in Poland and the Russian Far east indicate that the *A. sidemi* egg output and transmission are confined to the period from early summer to September. For example, there was a notable decline in adult numbers during autumn following post-mortem analysis, suggesting *A. sidemi* may preferentially survive the winter months as L4 larvae and juveniles (11, 20). In contrast, our results suggest that under the current Central European climate and management conditions at the study location, *A. sidemi* egg shedding may occur almost year-round from April to January. This finding is consistent with previous studies conducted in the CR that reported the occurrence of *A. sidemi* adults in wild ruminants and lack of hypobiosis during the winter (15, 21). A possible explanation for this shift could be recent higher temperatures that are more favorable for the parasite development than those prevailing during investigations of *A. sidemi* seasonal dynamics fifteen years ago or earlier. According to daily data collected from the European Climate Assessment & Dataset-ECA&D (47), the average minimum temperature in December at the location in our study was found to be 11.55°C higher compared to that of the Russian study in the Primorsky region between 1951 and 1955 (20) and on average 4.55°C higher than in the Polish study conducted between 1997 and 2001 (11). Furthermore, our study site experienced, on average, fewer frost days during December compared to the aforementioned studies. As modeled on the development cycle of the cervid trichostrongylid nematode *Ostertagia gruehneri*, a warmer climate may prolong the transmission of the parasite as mild temperatures facilitate both earlier and later larval development in spring and autumn, respectively (48). The climatic conditions experienced by the larvae during exogenous development are also one of the factors determining whether they undergo hypobiosis. In the present study, we found a significantly positive relationship between the mean temperature and the non-zero values of the targeted *A. sidemi* DNA, which suggests that changes in contamination levels are likely influenced by the environmental factors that impact parasite development and transmission. On the other hand, the low variability of positive values within a year may indicate a lack of seasonality of the parasite. Changes toward a shift from traditional seasonality to uniform through-year distribution of trichostrongylid infections associated with a warming climate have been reported on sheep farms in Northern Ireland (22). More recently, a similar pattern of year-round egg production with little seasonality has been observed in farmed deer and wapiti in New Zealand (25).

In this study, we investigated seasonal changes in the egg production of an invasive parasite, *A. sidemi*, measured through detection and quantification of target DNA from coprocultured L3 following monthly faecal sampling of fallow deer on a game reserve in the CR. The presence and amount of *A. sidemi* DNA detected was examined relative to environmental factors and anthelmintic treatment, but seasonal parasite dynamics is a complex phenomenon with many factors contributing to its outcome. The seasonal pattern of parasite egg shedding can vary between host sexes due to different behavior and timing of investment into reproduction which subsequently alter immunity to parasites. At the same time, it can vary depending on the age or nutritional status of the host. Due to the unavailability of individual metadata on the sampled animals in our study, we could not assess whether the seasonal dynamics of the parasite differed by host sex, age class, or individual condition. Further assessment of the development and survival of *A. sidemi* life stages must also be an important consideration in the design of future investigations to improve predictions of seasonal dynamics, and thus infection risk attributed to this parasite in time and space. Although our results shed light on seasonal dynamics of *A. sidemi* in the CR, it is important to acknowledge limitations arising from the relatively small number of animals we sampled. Future studies with appropriately estimated sample sizes are needed to understand the epidemiology of ashworthiosis more comprehensively.

## 5. Conclusion

This study provides new insight into the epidemiology of a non-native nematode in the changing world. Our findings indicate almost negligible seasonal parasite egg shedding patterns in the current climatic conditions of the CR, contrasting with previous studies conducted more than a decade ago. We strongly recommend regular assessment of *A. sidemi* infection in cloven-hoofed animals in game reserves using reliable molecular tools and to take appropriate control measures to prevent the spread of this parasite to wild animal populations. This approach is especially desirable in animal species sensitive to this infection and those kept in conservation programmes. The control of *A. sidemi* is challenging once the parasite is well established among animals/at the locality. The use of anthelmintic drugs has been shown to suppress egg shedding and thus further pasture contamination significantly; however, treatment efficacy relative to the absence of *A. sidemi* DNA in coprocultured L3 in the current study was relatively short-lived. For an improved understanding of *A. sidemi* seasonal dynamics and subsequently development of effective parasite control strategies, it is necessary to investigate the contributions of all cohorts within the herd (including age groups and sexes) to the overall pasture contamination throughout the year.

## Data availability statement

The raw data supporting the conclusions of this article will be made available by the authors, without undue reservation.

## Ethics statement

The requirement of ethical approval was waived by Institutional ethics and animal welfare committee of the Czech University of Life Sciences Prague for the studies involving animals because faecal samples were collected from the pasture without any direct contact with animals. The studies were conducted in accordance with the local legislation and institutional requirements. Written informed consent was not obtained from the owners for the participation of their animals in this study because the game reserve owner do not required written consent.

## Author contributions

JM: Data curation, Formal analysis, Methodology, Validation, Visualization, Writing – original draft. LŠ: Data curation, Formal analysis, Methodology, Writing – original draft. CM: Conceptualization, Data curation, Writing – review & editing. JV: Conceptualization, Funding acquisition, Project administration, Supervision, Writing – review & editing.

## References

[1] Esattore B Saggiomo L Sensi M Francia V Cherin M. Tell me what you eat and I’ll tell you…where you live: an updated review of the worldwide distribution and foraging ecology of the fallow deer (*Dama dama*). Mamm Biol. (2022) 102:321–38. doi: 10.1007/s42991-022-00250-6

[2] Kudrnáčová E Bartoň L Bureš D Hoffman LC. Carcass and meat characteristics from farm-raised and wild fallow deer (*Dama dama*) and red deer (*Cervus elaphus*): a review. Meat Sci. (2018) 141:9–27. doi: 10.1016/j.meatsci.2018.02.020, PMID: 29558697

[3] Bartoš L Kotrba R Pintíř J. Ungulates and their management in the Czech Republic In: Appolonio M Andersen R Putman R, editors. European ungulates and their management in the 21st century. Cambridge: Cambridge University Press (2010)

[4] Gunn A Irvine R. Subclinical parasitism and ruminant foraging strategies – a review. Wildl Soc Bull. (2003) 31:117–26. doi: 10.2307/3784365

[5] Dróżdż J Malczewski A Demiaszkiewicz A Lachowicz J. The helminthofauna of farmed deer (Cervidae) in Poland. Acta Parasitol. (1997) 42:225–9.

[6] Santín-Durán M Alunda JM Hoberg EP De La Fuente C. Abomasal parasites in wild sympatric cervids, red deer, *Cervus elaphus* and fallow deer, *Dama dama*, from three localities across central and Western Spain: relationship to host density and park management. J Parasitol. (2004) 90:1378–86. doi: 10.1645/GE-3376 15715232

[7] Rehbein S Visser M Jekel I Silaghi C. Endoparasites of the fallow deer (*Dama dama*) of the Antheringer au in Salzburg, Austria. Wien Klin Wochenschr. (2014) 126:37–41. doi: 10.1007/s00508-014-0506-8, PMID: 24535173

[8] Kowal J Nosal P Bonczar Z Wajdzik M. Parasites of captive fallow deer (*Dama dama* L.) from southern Poland with special emphasis on *Ashworthius sidemi*. Ann Parasitol. (2012) 58:23–6. 23094333

[9] Kuznetsov D. The first detection of Abomasal nematode *Ashworthius sidemi* in fallow deer (*Dama dama*) in Russia. Acta Parasitol. (2022) 67:560–3. doi: 10.1007/s11686-021-00452-x, PMID: 34263441

[10] Ferté H Cléva D Depaquit J Gobert S Léger N. Status and origin of Haemonchinae (Nematoda: Trichostrongylidae) in deer: a survey conducted in France from 1985 to 1998. Parasitol Res. (2000) 86:582–7. doi: 10.1007/PL00008534 10935910

[11] Dróżdż J Demiaszkiewicz A Lachowicz J. Expansion of the Asiatic parasite *Ashworthius sidemi* (Nematoda, Trichostrongylidae) in wild ruminants in polish territory. Parasitol Res. (2003) 89:94–7. doi: 10.1007/s00436-002-0675-7, PMID: 12489006

[12] Kuzmina T Kharchenko V Malega A. Helminth fauna of roe deer (*Capreolus Capreolus*) in Ukraine: biodiversity and parasite community. Vestn Zool. (2010) 44:e-12–9. doi: 10.2478/v10058-010-0002-1

[13] Kotrlá B Kotrlý A. Helminths of wild ruminants introduced into Czechoslovakia. Folia Parasitol (Praha). (1977) 24:35–40. PMID: 852770

[14] Demiaszkiewicz AW Merta D Kobielski J Filip KJ Pyziel AM. Expansion of *Ashworthius sidemi* in red deer and roe deer from the lower Silesian wilderness and its impact on infection with other gastrointestinal nematodes. Acta Parasitol. (2017) 62:853–7. doi: 10.1515/ap-2017-0103, PMID: 29035860

[15] Vadlejch J Kyriánová IA Rylková K Zikmund M Langrová I. Health risks associated with wild animal translocation: a case of the European bison and an alien parasite. Biol Invasions. (2017) 19:1121–5. doi: 10.1007/s10530-016-1306-z

[16] Belem AM Couvillion CE Siefker C Griffin RN. Evidence for arrested development of abomasal nematodes in white-tailed deer. J Wildl Dis. (1993) 29:261–5. doi: 10.7589/0090-3558-29.2.261 8487375

[17] O’Connor LJ Walkden-Brown SW Kahn LP. Ecology of the free-living stages of major trichostrongylid parasites of sheep. Vet Parasitol. (2006) 142:1–15. doi: 10.1016/j.vetpar.2006.08.035, PMID: 17011129

[18] Armour J. The epidemiology of helminth disease in farm animals. Vet Parasitol (1980) 6:7–46. doi: https://doi.org/10.1016/0304-4017(80)90037-0

[19] Charlier J Höglund J Morgan ER Geldhof P Vercruysse J Claerebout E. Biology and epidemiology of gastrointestinal nematodes in cattle. Vet Clin North Am Food Anim Pract. (2020) 36:1–15. doi: 10.1016/j.cvfa.2019.11.001, PMID: 32029177

[20] Ovcharenko DA. Seasonal dynamics and development of *Ashworthius sidemi* (Trichostrongylidae), *Oesophagostomum radiatum* and *O. Venulosum* (Strongylidae) of *Cervus nippon hortulorum*. Parazitologiya. (1968) 2:470–4.

[21] Magdálek J Bourgoin G Vadlejch J. Non-native nematode *Ashworthius sidemi* currently dominates the Abomasal parasite Community of Cervid Hosts in the Czech Republic. Front Vet Sci. (2022) 9:862092. doi: 10.3389/fvets.2022.862092, PMID: 35573405 PMC9096835

[22] McMahon C Gordon AW Edgar HWJ Hanna REB Brennan GP Fairweather I. The effects of climate change on ovine parasitic gastroenteritis determined using veterinary surveillance and meteorological data for Northern Ireland over the period 1999-2009. Vet Parasitol. (2012) 190:167–77. doi: 10.1016/j.vetpar.2012.06.016, PMID: 22789298

[23] Altizer S Ostfeld RS Johnson PTJ Kutz S Harvell CD. Climate change and infectious diseases: from evidence to a predictive framework. Science. (2013) 341:514–9. doi: 10.1126/science.1239401 23908230

[24] Pilarczyk B Tomza-Marciniak A Udała J Kuba J. The prevalence and control of gastrointestinal nematodes in farmed fallow deer (*Dama dama* L.). Vet Arh. (2015) 85:415–23. doi: 10.13140/RG.2.1.2340.5922

[25] Chambers A Candy P Green P Sauermann C Leathwick D. Seasonal output of gastrointestinal nematode eggs and lungworm larvae in farmed wapiti and red deer of New Zealand. Vet Parasitol. (2022) 303:109660. doi: 10.1016/j.vetpar.2022.109660, PMID: 35168114

[26] Pedersen AB Fenton A. The role of antiparasite treatment experiments in assessing the impact of parasites on wildlife. Trends Parasitol. (2015) 31:200–11. doi: 10.1016/j.pt.2015.02.004, PMID: 25778845

[27] Chroust K Vitula F. Anthelmintic efficacy of Cermix premix of the nematodes in game animals. Veterinarstvi. (2005) 55:707–13.

[28] Lamka J Simon I Čapková J Vysloužil L. Efficacy of two- and four-day treatments with Rafendazol premix Spofa in game animals. Veterinarstvi. (1990) 40:501–2.

[29] Lamka J Peška R Kulichová E Urešová J Vondřejc M. Anthelmintic efficacy of orally administered ivermectin against nematodes in the moufflon (*Ovis musimon*). Acta Vet Brno. (1996) 65:225–8. doi: 10.2754/avb199665030225

[30] Lichtenfels JR Hoberg EP Zarlenga DS. Systematics of gastrointestinal nematodes of domestic ruminants: advances between 1992 and 1995 and proposals for future research. Vet Parasitol. (1997) 72:225–45. doi: 10.1016/S0304-4017(97)00099-X 9460200

[31] Moskwa B Bień J Goździk K Cabaj W. The usefulness of DNA derived from third stage larvae in the detection of *Ashworthius sidemi* infection in European bison, by a simple polymerase chain reaction. Parasit Vectors. (2014) 7:215–5. doi: 10.1186/1756-3305-7-215 24886355 PMC4019785

[32] Reslová N Škorpíková L Kyriánová IA Vadlejch J Höglund J Skuce P. The identification and semi-quantitative assessment of gastrointestinal nematodes in faecal samples using multiplex real-time PCR assays. Parasit Vectors. (2021) 14:391. doi: 10.1186/s13071-021-04882-4, PMID: 34372893 PMC8351436

[33] Beck HE Zimmermann NE McVicar TR Vergopolan N Berg A Wood EF. Present and future Köppen-Geiger climate classification maps at 1-km resolution. Sci Data. (2018) 5:180214. doi: 10.1038/sdata.2018.214 30375988 PMC6207062

[34] Ahrens CD. Meteorology today: An introduction to weather, climate, and the environment. Belmont, CA: Cengage Learning Canada Inc (2015).

[35] Jørgen H Perry BD Brian D. The epidemiology, diagnosis, and control of helminth parasites of ruminants: A handbook. Nairobi: International Laboratory for Research on Animal Diseases (1994).

[36] Mikel P Vašíčková P Tesačík R Malenovská H Kulich P Veselý T. Preparation of MS2 phage-like particles and their use as potential process control viruses for detection and quantification of enteric RNA viruses in different matrices. Front Microbiol. (2016) 7:1911. doi: 10.3389/fmicb.2016.01911, PMID: 28133456 PMC5234545

[37] R Core Team. R: a language and environment for statistical computing. (2022) Available at: https://www.r-project.org/

[38] Wickham Hadley. ggplot2: Elegant graphics for data analysis. Springer-Verlag New York (2016). Available at: https://ggplot2.tidyverse.org

[39] Dróżdż J Lachowics J Demiaszkiewicz A. Seasonal changes in the helminth fauna of *Cervus elaphus* (L.) from Słowiński National Park (Poland). Acta Parasitol. (1993) 38:85–7.

[40] Albery GF Kenyon F Morris A Morris S Nussey DH Pemberton JM. Seasonality of helminth infection in wild red deer varies between individuals and between parasite taxa. Parasitology. (2018) 145:1410–20. doi: 10.1017/S0031182018000185, PMID: 29519265 PMC6137381

[41] Kołodziej-Sobocińska M Pyziel AM Demiaszkiewicz AW Borowik T Kowalczyk R. Pattern of parasite egg shedding by European bison (*Bison bonasus*) in the Białowieża primeval Forest, Poland. Mamm Res. (2016) 61:179–86. doi: 10.1007/s13364-016-0270-4

[42] Borkovcová M Langrová I Totková A. Endoparasitoses of fallow deer (*Dama dama*) in game-park in South Moravia (Czech Republic). Helminthologia. (2013) 50:15–9. doi: 10.2478/s11687-013-0102-x

[43] Coop RL Kyriazakis I. Nutrition–parasite interaction. Vet Parasitol. (1999) 84:187–204. doi: 10.1016/S0304-4017(99)00070-9, PMID: 10456415

[44] Crofton HD. Nematode parasite populations in sheep on lowland farms V. Further observations on the post-parturient rise and a discussion of its significance. Parasitology. (1958) 48:243–50. doi: 10.1017/S0031182000021211, PMID: 13600851

[45] Hayward AD Pilkington JG Wilson K McNeilly TN Watt KA. Reproductive effort influences intra-seasonal variation in parasite-specific antibody responses in wild Soay sheep. Funct Ecol. (2019) 33:1307–20. doi: 10.1111/1365-2435.13330

[46] Hamer K McIntyre J Morrison AA Jennings A Kelly RF Leeson S. The dynamics of ovine gastrointestinal nematode infections within ewe and lamb cohorts on three Scottish sheep farms. Prev Vet Med. (2019) 171:104752. doi: 10.1016/j.prevetmed.2019.104752, PMID: 31479849

[47] Klein Tank AMG Wijngaard JB Können GP Böhm R Demarée G Gocheva A. Daily dataset of 20th-century surface air temperature and precipitation series for the European climate assessment. Int J Climatol. (2002) 22:1441–53. doi: 10.1002/joc.773

[48] Molnár PK Kutz SJ Hoar BM Dobson AP. Metabolic approaches to understanding climate change impacts on seasonal host-macroparasite dynamics. Ecol Lett. (2013) 16:9–21. doi: 10.1111/ele.12022, PMID: 23157563

